# Recognizing the roles of primary health care in addressing non-communicable diseases in low- and middle-income countries: Lesson from COVID-19, implications for the future

**DOI:** 10.7189/jogh.11.03120

**Published:** 2021-11-13

**Authors:** Uday Narayan Yadav, Sabuj Kanti Mistry, Saruna Ghimire, Carmen Huckel Schneider, Lal Bahadur Rawal, Shambhu Prasad Acharya, Ben Harris-Roxas, Mark Fort Harris

**Affiliations:** 1 National Centre for Epidemiology and Population Health, Research School of Population Health, The Australian National University, Canberra, Australia, Sydney, Australia; 2 Centre for Primary Health Care and Equity, University of New South Wales, Sydney, Australia; 3 Centre for Research Policy and Implementation (CRPIN), Biratnagar, Nepal; 4 BRAC James P Grant School of Public Health, BRAC University, Dhaka, Bangladesh; 5 ARCED Foundation, Dhaka, Bangladesh; 6 Department of Sociology and Gerontology and Scripps Gerontology Center, Miami University, Oxford, Ohio, USA; 7 Menzies Centre for Health Policy and Economics, Faculty of Medicine and Health, Sydney School of Public Health, University of Sydney, Sydney, Australia; 8 School of Health Medical and Applied Sciences, College of Science and Sustainability, Central Queensland University, Sydney Campus, Australia; 9 World Health Organization, Geneva, Switzerland; 10 School of Population Health, Faculty of Medicine and Health, University of New South Wales, Sydney, Australia

Non-communicable diseases (NCDs), a leading cause of global morbidity and mortality, are key challenges to achieving the 2030 Sustainable Development Goals. Looking at trends over the years, the surge in NCDs is anticipated to continue, with the greatest impact on the poor and marginalized groups, mainly from low- and middle-income countries (LMICs) [1]. During the COVID-19 pandemic, people with one or more underlying NCDs were particularly vulnerable given the increased risk of severe disease and death [2]. Despite being vulnerable, people living with NCDs (PLWNCDs) faced challenges meeting their health care needs, attributed to the preventive measures against COVID-19 such as physical distancing and nationwide lockdowns and restrictions, which further exacerbated their physical and mental health outcomes [1]. COVID-19 disrupted the regular health services as the demand for acute care surged and strained the already weak public health system of many countries, especially in those hardest-hit by COVID-19, such as India, Nepal, Bangladesh, Brazil, Iran, and some other LMICs, stretching them beyond their capacity. In fact, the pandemic in some LMICs contributed to a near collapse of health service delivery [1]. Given their increased vulnerability to COVID-19, although PLWNCDs required greater support and care to manage their conditions than ever before, overwhelmed health care systems failed to meet their needs.

Recalling the old truism constructed by Winston Churchill, “*one should never let a crisis go to waste*,” there is an opportunity to learn from COVID-19, specifically with regards to the prevention and control of NCDs. In this paper, we first highlight the challenges faced by PLWNCDs during the COVID-19 pandemic, then discuss how primary health care (PHC) can act as a critical foundation for strategies to meet the health needs of the PLWNCDs during current and future outbreaks and emergencies.

**Figure:**
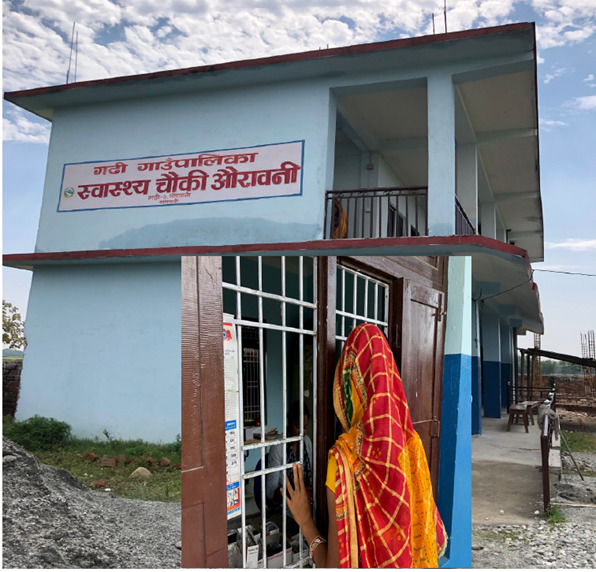
Photo: Peripheral health system of Nepal captured by Uday N Yadav in 2019 (used with permission).

## CHALLENGES FACED BY PEOPLE WITH NCDS IN THE COVID-19 PANDEMIC

As NCDs and COVID-19 are highly correlated, they cannot be addressed in siloes. Further, during outbreaks, health systems have dual responsibilities to respond to acute system demand as well as continuing to provide essential health services. However, a rapid assessment by the World Health Organization (WHO) on the impact of COVID-19 on NCD resources and services revealed a considerable disruption to NCD services in many LMICs due of a lack of budget allocation for NCDs by governments or relocation of funds from NCDs to COVID-19 management [3]. Additionally, LMICs were much less likely than high-income countries to include NCDs in their COVID-19 response plans [4].

Despite the importance of prevention and control of NCDs during the pandemic [5], preventive measures such as lockdowns have made health care almost inaccessible [6]. Public transport, the main means of transport for low-socioeconomic groups, ceased in many LMICs such as Nepal, India, Bangladesh during the COVID-19 pandemic. As private transport is accessible only to the wealthier groups in many of these countries [7,8], these restrictions disrupted visits to health facilities and/or seeking health care for acute care and essential services related to NCDs. Several studies from LMICs also revealed severe disruption of health services for PLWNCDs, and consequently, the need for alternative delivery methods to face-to-face consultations [9-11]. Moreover, fear of contracting COVID-19 while seeking treatment also jeopardized their access to care [12].

Emerging evidence from LMICs also shows that PLWNCDs, particularly from marginalized populations, experience myriad challenges in accessing health services, and procuring medication for pre-existing conditions, missed follow-up visits, and experienced worsening of their pre-existing conditions [4]. The lockdown measures also aggravated risk factors for NCDs such as a sedentary lifestyle, unhealthy diet/less access to nutritional foods as well as increased smoking, alcohol and tobacco use [1,11].

## PRIMARY HEALTH CARE AND ITS ROLE IN PREVENTION AND CONTROL OF NCDS IN LMICS

Recognizing the global burden and impact of NCDs on individuals and societies, WHO developed the Global Strategy for Prevention and Control of NCDs (2013-2020) and Package of Essential Noncommunicable (PEN) Disease Interventions for integration and management of NCDs into PHC in low-resource settings, which has been endorsed by 193 member states [13]. In any public health emergency, PHC can play a crucial role in addressing the emergency-introduced local public health needs within the limit of available resources. However, a survey conducted by WHO reported that NCDs were not prioritized in the COVID-19 response plans of many LMICs [3]. In the current pandemic, many health leaders and policymakers of LMICs have failed to consider PHC as an essential part of the COVID-19 response, which created constraints on secondary and tertiary health facilities.

Despite the need to establish a strong PHC at the community/population level, LMICs, often with the assistance of donor nations/organizations, have focused on establishing teaching hospitals, medical and nursing schools, and disease-specific vertical programs [14,15]. LMICs do have the concept of strong PHC incorporated in their plans and policies. However, in reality, their PHC services are not in a position to deliver comprehensive health care services. Often a major portion of the budget is invested in secondary and tertiary hospitals and there is a lack of linkage between the overall health plan and its actual implementation in LMICs. Tertiary-level health services are expensive and concentrated in urban areas, making access to comprehensive health care challenging for rural populations.

## WAYS FORWARD

**Figure 1:**
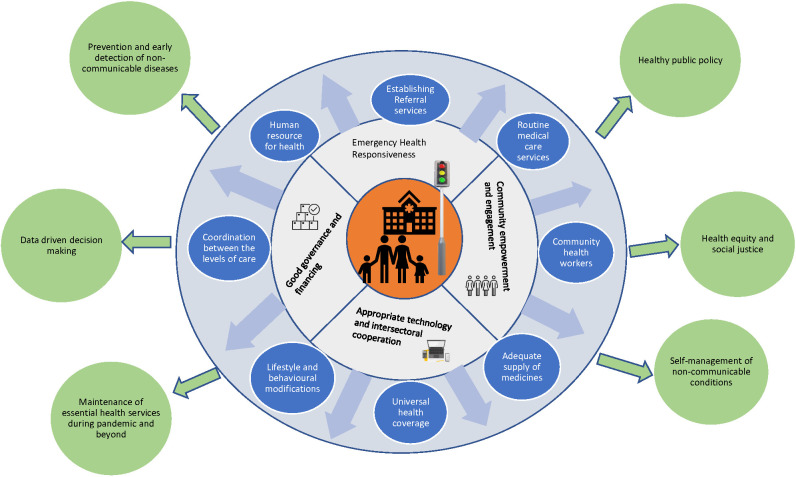
Comprehensive care framework for people with noncommunicable diseases using primary health care (PHC) approach. White circle: Pillars of PHC. Purple circle: Elements of PHC. Green circle: Benefits of comprehensive preventive and promotive health care services for people with non-communicable disease.

During and beyond the pandemic, continuity of comprehensive care for PLWNCDs can be delivered through a strong PHC system by engaging community health workers in promoting self-management of NCDs, expanding digital innovation at the PHC level, and introducing policies, advocacy, and research to broaden the scope and our knowledge of NCDs management through PHC. **Figure 1** demonstrates the essential components/elements of PHC required for required for addressing health needs of PLWNCDs during pandemic and beyond.

### Revitalization of PHC

Strong PHC systems are essential to maintaining essential health care services in communities, but the workforce shortages and absenteeism have hindered the provision of care through PHC. Specifically, in LMICs, the availability of a health workforce at the PHC level is substantially below the level recommended by WHO [16]. Mixed interventions, such as free or subsidized educational and training opportunities for students from rural, indigenous tribes and marginalized communities, providing incentives to health care staff to move to rural areas of shortage, and re-locating staffs in their own local bodies or province have been adopted by various countries to address the issues of health workforce shortage [17,18]. During COVID-19 pandemic, PHC has been mobilized in LMICs for continuity of care for PLWNCDs by re-organizing service delivery for better access (eg, expanding home-based care and provision of virtual counseling), operating NCD clinics [12], involving community health workers in delivery of medicines, extending the operation of pharmacies and PHC to 24 hours to maintain essential services and operating teleconsultation services [19,20]. In Thailand, access to medicines for PLWNCDs was maintained through village health volunteers or postal services (17). Drawing upon these leanings, it is vital to involve PHC in emergency preparedness and developing strategies to provide health services to vulnerable people like PLWNCDS. The pandemic had demonstrated that clear lines of responsibility should be given to PHC for ensuring continuity of care for vulnerable groups such as PLWNCDs.

### Self-management support and engagement of community health workers

As treatment of NCDs can be expensive, it may pose major economic challenges to health care systems in many LMICs. Hence, comprehensive self-management that can reduce health care expenses [21], should be prioritized and expanded in LMICs. Community health workers (CHWs) are a well-established, effective, and relatively inexpensive human health resource in many LMICs for delivering self-management support for chronic disease patients [22]. CHW’s involvement in PHC delivery saves time and financial cost without compromising the quality of care or health outcomes for patients [23]. In this context, CHWs can be trained to empower and engage PLWNCDs to prevent and manage NCDs in LMICs during the COVID-19 pandemic and beyond.

### Opportunity for digital innovation at the level of PHC

Although telemedicine existed pre-pandemic, it received significant attention in the COVID-19 era as an effective alternative for health service delivery when face-to-face consultations are not feasible [24]. Some LMICs such as Egypt, Pakistan, India, Philippines, Sri Lanka, China etc. have leveraged telemedicine to deliver health care services for people with NCDs during COVID-19 pandemic. For example, the “e-sanjeevani” initiative by the Government of India has been used to monitor symptoms and provide advice on self-care for PLWNCDs [19,25]. The COVID-19 pandemic is a wake-up call for policy makers of LMICs to frame regulations and standards to support the adoption of telemedicine to help and alleviate the pressure on health care systems during and beyond the pandemic.

### Policy, advocacy, and research

In general, and specifically during this pandemic, emergency management policies were often centered on hospitals rather than PHC. This highlights the need to incorporate PHC in all policies, strategies, and services developed to manage emergencies. During the current and future pandemics, PHC could be leveraged as a critical foundation and the first line of defense for direct surveillance and management of outbreaks through community testing, contact tracing, outbreak communication, isolation, and other public health and social measures that have been crucial in slowing down disease transmission as well as a service delivery mechanism for the vulnerable population such as PLWNCDS. Policymakers and health care leaders also need to prioritize investment in human health resources in PHC as well as the essential diagnostic and medical supplies required for PHC to deliver comprehensive PHC services.

Researchers working in LMICs have the opportunity to undertake studies on: (i) documenting their experience of implementing PEN package at PHC during the pandemic (ii) design and evaluate the acceptability and effectiveness of digital health technologies in the management of NCDs within PHC, and (ii) design and test models of care with PHC at the frontline of holistic care for PLWNCDs.

## CONCLUSION

The COVID-19 pandemic has impacted the lives of all, including PLWNCDs. The public health actions implemented by the governments of LMICs during the COVID-19 pandemic have been largely directed towards strengthening secondary and tertiary care and containment strategies for COVID-19 while continuity of care for PLWNCDs did not receive enough attention. PHC could be strengthened to blunt the impact of the current and future pandemics and emergencies on public health by providing continuity of care and essential health services to vulnerable and disadvantage groups. Some ideas to strengthen PHC includes delivering comprehensive preventive and treatment services linked to other levels of care, innovating PHC delivery through the use of digital technology, mobilization of CHWs to provide to localized health care both during the COVID-19 pandemic and beyond.

## References

[1] Yadav UN Rayamajhee B Mistry SK Parsekar SS Mishra SK A Syndemic Perspective on the Management of Non-communicable Diseases Amid the COVID-19 Pandemic in Low- and Middle-Income Countries. Front Public Health. 2020;8:508. 10.3389/fpubh.2020.00508 33102414 PMC7545493

[2] The Lancet COVID-19: a new lens for non-communicable diseases. Lancet. 2020;396:649. 10.1016/S0140-6736(20)31856-0 32891195 PMC7470688

[3] World Health Organization. The impact of the COVID-19 pandemic on noncommunicable disease resources and services: results of a rapid assessment. Geneva: WHO; 2020.

[4] Singh K Kondal D Mohan S Jaganathan S Deepa M Venkateshmurthy NS Health, psychosocial, and economic impacts of the COVID-19 pandemic on people with chronic conditions in India: a mixed methods study. BMC Public Health. 2021;21:685. 10.1186/s12889-021-10708-w 33832478 PMC8027966

[5] Wang B Li R Lu Z Huang Y Does comorbidity increase the risk of patients with COVID-19: evidence from meta-analysis. Aging (Albany NY). 2020;12:6049-57. 10.18632/aging.103000 32267833 PMC7185114

[6] Pati S Mahapatra P Kanungo S Uddin A Sahoo KC Managing Multimorbidity (Multiple Chronic Diseases) Amid COVID-19 Pandemic: A Community Based Study From Odisha, India. Front Public Health. 2021;8:584408. 10.3389/fpubh.2020.584408 33598442 PMC7882709

[7] Paul B, Sarkar S. The Contagion Effects of COVID-19 and Public Transportation System: Conceptualizing the Shifting Paradigm in India: COVID-19 Pandemic Trajectory in the Developing World. AGES. Springer, Singapore 2020 Dec 29:231-55. eCollection 2021.

[8] O’Toole R. Reimagining Transportation Policy During And After Covid-19. Reason Foundation. 2020. Available: https://reason.org/policy-brief/reimagining-transportation-policy-during-and-after-covid-19/. Accessed: 20 May 2021.

[9] Chudasama YV Gillies CL Zaccardi F Coles B Davies MJ Seidu S Impact of COVID-19 on routine care for chronic diseases: A global survey of views from healthcare professionals. Diabetes Metab Syndr. 2020;14:965-7. 10.1016/j.dsx.2020.06.042 32604016 PMC7308780

[10] Kendzerska T Zhu DT Gershon AS Edwards JD Peixoto C Robillard R The effects of the health system response to the COVID-19 pandemic on chronic disease management: a narrative review. Risk Manag Healthc Policy. 2021;14:575. 10.2147/RMHP.S293471 33623448 PMC7894869

[11] Leite JS Feter N Caputo EL Doring IR Cassuriaga J Reichert FF Managing noncommunicable diseases during the COVID-19 pandemic in Brazil: findings from the PAMPA cohort. Cien Saude Colet. 2021;26:987-1000. 10.1590/1413-81232021263.39232020 33729353

[12] Mistry SK Ali ARMM Akther F Yadav UN Harris MF Exploring fear of COVID-19 and its correlates among older adults in Bangladesh. Global Health. 2021;17:47. 10.1186/s12992-021-00698-0 33853616 PMC8045579

[13] Allen LN Nicholson BD Yeung BYT Goiana-da-Silva F Implementation of non-communicable disease policies: a geopolitical analysis of 151 countries. Lancet Glob Health. 2020;8:e50-8. 10.1016/S2214-109X(19)30446-2 31813787 PMC7024987

[14] World Health Assembly. Strengthening the capacity of governments to constructively engage the private sector in providing essential health-care services. Geneva: World Health Organization; 2010.

[15] Vande Maele N Xu K Soucat A Fleisher L Aranguren M Wang H Measuring primary healthcare expenditure in low-income and lower middle-income countries. BMJ Glob Health. 2019;4:e001497. 10.1136/bmjgh-2019-001497 30997157 PMC6441277

[16] Mills A Health Care Systems in Low- and Middle-Income Countries. N Engl J Med. 2014;370:552-7. 10.1056/NEJMra1110897 24499213

[17] Tangcharoensathien V Deep impacts of COVID-19: overcoming challenges in strengthening primary health care by targeting the health workforce. WHO-SEAJPH. 2021;10:73-5.

[18] World Health Organization. The Decade for Health Workforce Strengthening in the SEA Region 2015–2024: mid-term review of progress, 2020. New Delhi: World Health Organization. Regional Office for South-East Asia; 2019.

[19] Okoro RN COVID-19 pandemic: The role of community pharmacists in chronic kidney disease management supportive care. Res Social Adm Pharm. 2021;17:1925-8. 10.1016/j.sapharm.2020.07.008 33317766 PMC7341043

[20] Gummidi B John O Jha V Continuum of care for non-communicable diseases during COVID-19 pandemic in rural India: A mixed methods study. J Family Med Prim Care. 2020;9:6012-7. 10.4103/jfmpc.jfmpc_1805_20 33681035 PMC7928131

[21] Basu R Ory MG Towne SD Jr Smith ML Hochhalter AK Ahn S Cost-effectiveness of the chronic disease self-management program: implications for community-based organizations. Front Public Health. 2015;3:27. 10.3389/fpubh.2015.00027 25964945 PMC4410335

[22] Werfalli M Raubenheimer PJ Engel M Musekiwa A Bobrow K Peer N The effectiveness of peer and community health worker-led self-management support programs for improving diabetes health-related outcomes in adults in low- and-middle-income countries: a systematic review. Syst Rev. 2020;9:133. 10.1186/s13643-020-01377-8 32505214 PMC7275531

[23] Vaughan K Kok MC Witter S Dieleman M Costs and cost-effectiveness of community health workers: evidence from a literature review. Hum Resour Health. 2015;13:71. 10.1186/s12960-015-0070-y 26329455 PMC4557864

[24] Monaghesh E Hajizadeh A The role of telehealth during COVID-19 outbreak: a systematic review based on current evidence. BMC Public Health. 2020;20:1193. 10.1186/s12889-020-09301-4 32738884 PMC7395209

[25] Gupta SK Lakshmi PVM Kaur M Rastogi A Role of self-care in COVID-19 pandemic for people living with comorbidities of diabetes and hypertension. J Family Med Prim Care. 2020;9:5495-501. 10.4103/jfmpc.jfmpc_1684_20 33532385 PMC7842493

